# The potential of GPT-4 advanced data analysis for radiomics-based machine learning models

**DOI:** 10.1093/noajnl/vdae230

**Published:** 2024-12-23

**Authors:** Martha Foltyn-Dumitru, Aditya Rastogi, Jaeyoung Cho, Marianne Schell, Mustafa Ahmed Mahmutoglu, Tobias Kessler, Felix Sahm, Wolfgang Wick, Martin Bendszus, Gianluca Brugnara, Philipp Vollmuth

**Affiliations:** Division for Computational Radiology & Clinical AI (CCIBonn.ai), Department of Neuroradiology, Bonn University Hospital, Bonn, Germany; Division for Computational Neuroimaging, Heidelberg University Hospital, Heidelberg, Germany; Department of Neuroradiology, Heidelberg University Hospital, Heidelberg, Germany; Division for Computational Radiology & Clinical AI (CCIBonn.ai), Department of Neuroradiology, Bonn University Hospital, Bonn, Germany; Division for Computational Neuroimaging, Heidelberg University Hospital, Heidelberg, Germany; Department of Neuroradiology, Heidelberg University Hospital, Heidelberg, Germany; Division for Computational Radiology & Clinical AI (CCIBonn.ai), Department of Neuroradiology, Bonn University Hospital, Bonn, Germany; Division for Computational Neuroimaging, Heidelberg University Hospital, Heidelberg, Germany; Department of Neuroradiology, Heidelberg University Hospital, Heidelberg, Germany; Division for Computational Neuroimaging, Heidelberg University Hospital, Heidelberg, Germany; Department of Neuroradiology, Heidelberg University Hospital, Heidelberg, Germany; Division for Computational Neuroimaging, Heidelberg University Hospital, Heidelberg, Germany; Department of Neuroradiology, Heidelberg University Hospital, Heidelberg, Germany; Clinical Cooperation Unit Neurooncology, German Cancer Research Center (DKFZ), Heidelberg, Germany; Department of Neurology and Neurooncology Program, Heidelberg University Hospital, Heidelberg University, Heidelberg, Germany; Clinical Cooperation Unit Neuropathology, German Cancer Consortium (DKTK), German Cancer Research Center (DKFZ), Heidelberg, Germany; Department of Neuropathology, Heidelberg University Hospital, Heidelberg, Germany; Clinical Cooperation Unit Neurooncology, German Cancer Research Center (DKFZ), Heidelberg, Germany; Department of Neurology and Neurooncology Program, Heidelberg University Hospital, Heidelberg University, Heidelberg, Germany; Department of Neuroradiology, Heidelberg University Hospital, Heidelberg, Germany; Division for Medical Image Computing (MIC), German Cancer Research Center (DKFZ), Heidelberg, Germany; Division for Computational Radiology & Clinical AI (CCIBonn.ai), Department of Neuroradiology, Bonn University Hospital, Bonn, Germany; Division for Computational Neuroimaging, Heidelberg University Hospital, Heidelberg, Germany; Department of Neuroradiology, Heidelberg University Hospital, Heidelberg, Germany; Division for Medical Image Computing (MIC), German Cancer Research Center (DKFZ), Heidelberg, Germany; Division for Computational Radiology & Clinical AI (CCIBonn.ai), Department of Neuroradiology, Bonn University Hospital, Bonn, Germany; Division for Computational Neuroimaging, Heidelberg University Hospital, Heidelberg, Germany; Department of Neuroradiology, Heidelberg University Hospital, Heidelberg, Germany

**Keywords:** glioma, IDH, large language models, machine learning, radiomics

## Abstract

**Background:**

This study aimed to explore the potential of the Advanced Data Analytics (ADA) package of GPT-4 to autonomously develop machine learning models (MLMs) for predicting glioma molecular types using radiomics from MRI.

**Methods:**

Radiomic features were extracted from preoperative MRI of *n* = 615 newly diagnosed glioma patients to predict glioma molecular types (IDH-wildtype vs IDH-mutant 1p19q-codeleted vs IDH-mutant 1p19q-non-codeleted) with a multiclass ML approach. Specifically, ADA was used to autonomously develop an ML pipeline and benchmark performance against an established handcrafted model using various MRI normalization methods (N4, Zscore, and WhiteStripe). External validation was performed on 2 public glioma datasets D2 (*n* = 160) and D3 (*n* = 410).

**Results:**

GPT-4 achieved the highest accuracy of 0.820 (95% CI = 0.819-0.821) on the D3 dataset with N4/WS normalization, significantly outperforming the benchmark model’s accuracy of 0.678 (95% CI = 0.677-0.680) (*P* < .001). Class-wise analysis showed performance variations across different glioma types. In the IDH-wildtype group, GPT-4 had a recall of 0.997 (95% CI = 0.997-0.997), surpassing the benchmark’s 0.742 (95% CI = 0.740-0.743). For the IDH-mut 1p/19q-non-codel group, GPT-4’s recall was 0.275 (95% CI = 0.272-0.279), lower than the benchmark’s 0.426 (95% CI = 0.423-0.430). In the IDH-mut 1p/19q-codel group, GPT-4’s recall was 0.199 (95% CI = 0.191-0.206), below the benchmark’s 0.730 (95% CI = 0.721-0.738). On the D2 dataset, GPT-4’s accuracy was significantly lower (*P* < .001) than the benchmark’s, with N4/WS achieving 0.668 (95% CI = 0.666-0.671) compared with 0.719 (95% CI = 0.717-0.722) (*P* < .001). Class-wise analysis revealed the same pattern as observed in D3.

**Conclusions:**

GPT-4 can autonomously develop radiomics-based MLMs, achieving performance comparable to handcrafted MLMs. However, its poorer class-wise performance due to unbalanced datasets shows limitations in handling complete end-to-end ML pipelines.

**Key Points**

- GPT-4 can be used for easy online modeling of radiomic features.
- GPT-4 matches handcrafted models in classifying glioma types from MRI radiomics.
- Class imbalances were challenging with GPT-4’s less effective approach.

**Importance of the Study**

This study elucidates the potential of large language models, such as GPT-4, to autonomously develop machine learning (ML) models for medical applications, specifically focusing on radiomics from preoperative MRI for glioma classification. By demonstrating that GPT-4 can achieve performance comparable to that of handcrafted models, this research highlights an opportunity to democratize ML in clinical settings. This advancement enables clinicians to leverage sophisticated ML tools effectively, regardless of their technical expertise. Furthermore, the study addresses performance variations across different glioma types, underscoring the necessity for continued refinement in managing class imbalances to enhance the reliability and accuracy of these autonomous models.

Machine learning (ML) is at the forefront of driving innovations in artificial intelligence (AI), with large potential to reshape various aspects of medical research and clinical practices.^1,2^ ML models play a crucial and growing role in diverse sectors within healthcare, including imaging diagnostics, public health, and epidemiology, evaluation of clinical trials, and the organization of healthcare services.^2^ However, the technical expertise in developing, implementing, and validating effective models might limit their access to clinicians.^3^

Recent research underscores the profound impact of large language models (LLMs) like GPT-4 by OpenAI in radiology. These models demonstrate a robust capability to enhance radiological practices by accurately identifying prevalent errors in radiology reports,^4^ extracting oncological phenotypes from lung cancer CT reports,^5^ gathering procedural information from neuroradiology reports related to mechanical thrombectomy for ischemic stroke patients,^6^ and even achieving performance levels comparable to radiology board examination criteria without specific training in the field.^7^ The integration of LLMs into radiological workflows has the potential to decrease work hours and reduce operational costs while preserving report accuracy. Additionally, Tayebi Arasteh et al. explored the capabilities of ChatGPT Advanced Data Analysis (ADA)—an extension of GPT-4—in autonomously developing ML models using real-world datasets from clinical trials. These models, which predict outcomes like biomarker presence, cancer progression, and disease complications, perform comparably to those developed manually.^8^

Here, we aim to explore the utility and performance of GPT-4 with its ADA capability to construct ML models from high-dimensional radiomics data autonomously. Radiomics transforms medical imaging into high-dimensional data, enabling the extraction of quantitative features.^9^ These features, including shape, texture, and intensity metrics, offer insights beyond visual assessment, providing a noninvasive means to characterize diseases. ML plays a pivotal role in radiomics by facilitating the analysis of these high-dimensional datasets. Traditional ML methods, such as support vector machines (SVMs), random forests, and logistic regression, have been widely used to identify relationships between radiomic features and clinical endpoints.^10,11^ These clinical endpoints encompass survival prediction as well as the identification of genetic alterations.^12–14^ However, the lack of standardized definitions has so far hampered the clinical use of radiomics,^15^ and current studies rely on handcrafted ML models, which require prior training, resources, and guidance in ML theory and practice to reach state-of-the-art performance. We hypothesize that GPT-4 with its ADA capability might allow the construction of radiomics-based ML models autonomously with comparable accuracy to handcrafted ML models. For this purpose, we develop and validate GPT-based ML models to classify glioma molecular subtypes in a large multicentric dataset. Additionally, we examine the influence of intensity normalization on the performance of these models. Our analysis includes a comparative evaluation of the autonomous models against a handcrafted ML algorithm for glioma subtype prediction,^16^ providing insights into the capabilities and effectiveness of LLMs to bridge the gap between ML developers and clinicians.

## Materials and Methods

The study received approval from the institutional review board, exempting it from the requirement for informed consent (S-784 2018).

### Dataset and Image Acquisition

Adult patients diagnosed with glioma as per the WHO 2021 Classification who underwent preoperative MRI scans at the executing institute from March 2009 to July 2020 were retrospectively enrolled in the study (*n* = 621). This dataset is called D1 in the following for reasons of blinding. Every patient had available information on IDH and 1p/19q status, derived from DNA methylation assays.^17^ Based on these data, the gliomas were classified into 3 categories: IDH-wildtype (IDH-wt), IDH-mutant with 1p/19q-codeletion (IDH-mut codel), and IDH-mutant without 1p/19q-codeletion (IDH-mut non-codel). Due to poor MRI image quality, such as motion artifacts, *n* = 3 (0.48%) patients were excluded. Additionally, *n* = 3 (0.48%) patients were removed from the analysis owing to data processing errors, resulting in a final count of *n* = 615 (99.03%) patients in the study cohort.

The MRI scans were conducted using a 3T MRI (Magnetom Verio, Trio TIM, or Skyra by Siemens Healthcare) as part of standard clinical evaluations. The imaging protocol adhered to global standards,^18^ incorporating 3D T1-weighted images both pre- and post-contrast (0.1 mmol/kg of gadoterate meglumine) and axial 2D-FLAIR and T2-weighted images. Further details on the MRI parameters are provided in Supplementary Methods and Materials.

#### External Testing

For external testing, the study utilized 2 publicly accessible preoperative MRI glioma datasets, which, for reasons of blinding, are referred to in the following only as dataset 2 (D2) and dataset 3 (D3). D2, initially included *n* = 242 patients, and D3 initially included *n* = 501 patients. After quality and data integrity assessments, D2 was adjusted to *n* = 160 (66.12%) patients due to the exclusion of *n* = 41(16.94%) cases with poor MRI quality, *n* = 29 (11.98%) cases with unresolved IDH or 1p/19q status, and *n* = 12 (4.96%) cases due to unsuccessful intensity normalization. D3 ultimately comprised *n* = 410 (81.84%) patients, following the exclusion of *n* = 91(18.16%) cases that lacked information on 1p/19q status. Like the training dataset, the external testing datasets also contained T1-weighted images before and after administering contrast, along with FLAIR and T2-weighted images. All 3 datasets have already been used to the same extent in the benchmark model.^16^

**Figure 1.**
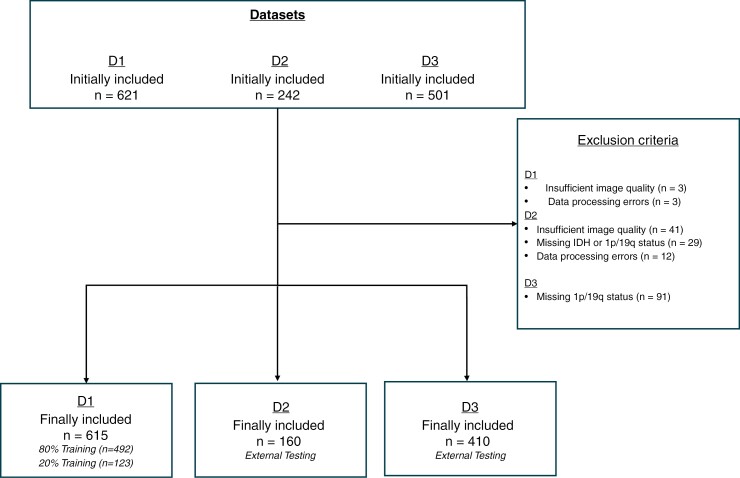
Flowchart of patient inclusion and exclusion criteria for the training and test datasets.

A flow diagram illustrating the composition of the study cohort is depicted in Figure 1.

### Image Processing and Radiomic Features Extraction

The images from D1 and D2 were analyzed using established, open-source software as detailed in earlier works.^19,20^ The processing pipeline comprised several sequential steps: (1) extraction of the brain tissue using the neural network-based HD-BET tool, accessible via https://github.com/MIC-DKFZ/HD-BET,^21^ (2) alignment of the image to the native T1-weighted image via rigid registration employing FSL (FMRIB’s Software Library), and (3) segmentation of tumor regions into contrast-enhancing, T2-FLAIR, and necrotic components using an adapted nnUNET version of the HD-GLIO algorithm (https://github.com/NeuroAI-HD/HD-GLIO).^19^ After segmentation, each was manually inspected and adjusted as needed by MF, a neuroradiology resident with 6 years of experience. D3 included preprocessed imaging sequences and a detailed tumor segmentation that identified different tumor compartments—enhancing, T2/FLAIR hyperintense, and non-enhancing/necrotic.

In our study, we assessed 4 distinct methods for intensity normalization, as described in the previous paper^16^: no normalization (naive), N4-bias-field-correction alone (N4), N4-bias-field-correction followed by Zscore normalization (N4/Zscore), and N4-bias-field-correction followed by WhiteStripe normalization (N4/WS).^22^ The implementations of this approach were carried out using the ANTsR and WhiteStripe software packages available in R (version 4.0.2, R Foundation for Statistical Computing, available at https://github.com/muschellij2/WhiteStripe).

#### Radiomic Features Extraction

Radiomic extraction and selection were conducted using Python (version 3.8.5) and the PyRadiomics library (https://pyradiomics.readthedocs.io).^23^ Adhering to the protocols established by the Image-Biomarker-Standardisation-Initiative (IBSI), we calculated all reproducible radiomic features across the utilized anatomical sequences from a whole tumor mask.^24^ The analysis included various feature categories: 13 shape-based, 17 first-order, 23 from the Gray-Level Co-occurrence Matrix (GLCM), 16 from the Gray-Level Run Length Matrix (GLRLM), 16 from the Gray-Level Size Zone Matrix (GLSZM), 14 from the Gray-Level Dependence Matrix (GLDM), and 5 from the Neighbouring Gray-Tone Difference Matrix (NGTDM). As the morphological radiomics remained consistent across all sequences, shape features were exclusively extracted from the T1 pre-contrast sequence. This comprehensive approach resulted in the extraction of 377 distinct radiomic features for each method of intensity normalization across each dataset.

### ML Model Development

D1 was partitioned into training and test subsets following an 80:20 ratio for the study. This division ensured that the representation of the 3 categories remained consistent in both subsets. D2 and D3 were used for external testing.

#### Benchmark Model

Based on previous findings where we applied the same dataset to evaluate the impact of different normalization methods on the predictive accuracy of various ML models for radiomics-based prediction of molecular glioma types we used a previously published SVM model as a benchmark.^16^ This model was developed using the scikit-learn-library in Python (version 3.8.5). To minimize bias in evaluating different normalization methods, we refrained from hyperparameter optimization, such as grid search, and adhered to the model’s default settings. The class imbalance was addressed by undersampling the IDH-wt group and employing the Synthetic Minority-Over-sampling Technique (SMOTE) [https://dl.acm.org/doi/10.5555/1622407.1622416] on the IDH-mut groups to achieve equal representation of call classes in the training dataset. We applied ANOVA *F*-statistics for feature selection to minimize overfitting and confounding effects, aiming to improve the signal-to-noise ratio. To follow a rule-of-thumb of approximately 100 cases per feature,^25^ we selected the 5 highest ranking radiomic features based on their *F*-statistic scores for each normalization approach, given our training set of 492 cases. The code will be published on our GitHub page after acceptance.

#### GPT-4 Model

The most recent version of GPT-4 was utilized online between February and March 2024 (https://chat.openai.com/).^26^ ADA was already integrated into this version and did not have to be activated as in previous versions. This version of GPT-4 operates within an integrated Python environment, employing Python 3.11.8. The radiomic dataset was supplied in an Excel format (Microsoft Corporation). Interaction with GPT-4 was initiated by outlining the study’s background, objectives, and dataset details, followed by instructions to develop, refine, and implement the most suitable ML model aligned with the study’s framework and generating predictions for glioma class, expressed as classification probabilities, without access to the ground truth. Each prompt segment was designed to be as neutral and precise as possible, with instructions focused on procedural steps rather than suggestive phrasing that might imply specific outcomes. The prompt to instruct GPT-4 is shown as a screenshot in Supplementary Figure 3. To mitigate bias compared with the benchmark model, the D1 dataset included a specific column indicating which patients were designated for training and which for testing. We provided GPT-4 with a comprehensive overview of the dataset. We specified that the dataset consisted of radiomic features extracted from MRI sequences and that the goal was to predict glioma types based on these features. We explicitly defined key columns, including: “multiclass” as the target variable indicating glioma type, “Patient_ID” as the identifier for unique patients, and “Split” to differentiate between training and internal test sets.

**Figure 2.**
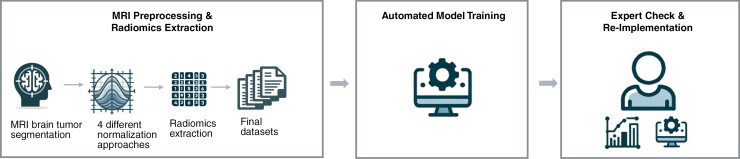
Workflow of the study. This workflow includes (1) MRI preprocessing and radiomics extraction, starting with MRI brain tumor segmentation, followed by application of 4 normalization approaches and extraction of radiomic features, leading to the compilation of the final datasets; (2) automated model training using GPT-4, where the model autonomously selects and trains algorithms; and (3) expert verification and reimplementation of the model to validate the approach and ensure robustness of the findings as well as benchmarking to handcrafted models. These icons were created with the assistance of DALL·E (OpenAI, February 2024).

By clarifying the dataset’s structure and terminology, we sought to reduce GPT-4’s interpretive bias by preventing it from making assumptions about data organization. In guiding GPT-4 toward enhanced predictive accuracy, we refrained from providing explicit ML advice, allowing the AI to independently select the most appropriate ML model for the dataset and generate predictions for the testing phase. After completing the model development, we instructed GPT-4 to save the results in CSV format and to provide the utilized Python code as a text file. This code was reviewed and reimplemented by A.R., a data scientist with 6 years of experience. GPT trained a Random Forest Classifier. It utilized class weights to manage the class imbalance because the SMOTE package was unavailable in this Python environment, as indicated by GPT. A graphic representation of the study design is shown in Figure 2. The icons of Figure 2 were created with the assistance of DALL·E (OpenAI, February 2024).

### Statistical Analysis

Models were evaluated using accuracy (=micro accuracy), macro average class accuracy (macro accuracy), weighted *F*1-score, macro average *F*1-score, weighted specificity, and macro average specificity as performance metrics. Micro accuracy calculates the overall metric by aggregating contributions from all classes. In contrast, macro accuracy computes the metric separately for each class and then averages these values, giving equal weight to each class. The class-specific evaluation was performed using *F*1-score, specificity, precision, and recall. 95% CIs were calculated using bootstrapping with *n* = 1000. To compare the accuracy and macro accuracy between the GPT-4 developed Random Forest Classifier and the handcrafted SVM model we conducted a paired 2-tailed student’s *t*-test. A *P*-value <.05 was considered significant for all analyses.

## Results

D1 comprised *n* = 442 (71.9%) IDH-wt *n* = 89 (14.5%) IDH-mut non-codel gliomas, as well as *n* = 84 (13.6%) IDH-mut codel gliomas. The external test datasets were composed as follows: *n* = 101 (63.13%) or *n* = 311 (75.85%) of IDH-wt, *n* = 42 (26.25%) or *n* = 84 (20.49%) of IDH-mut 1p/19q-non-codel, and *n* = 17 (10.63%) or *n* = 15 (3.66%) of IDH-mut 1p/19q-codel patients, across both D2 and D3, respectively. The demographic specifications of the included patients from all datasets are summarized in Table 1.

**Table 1.** Characteristics of the Study Population

		D1	D2	D3
Total no. of patients		615	160	410
Sex [*n* (%)]	Female	271 (44)	75 (47)	172 (42)
Age [y]	Mean ± SD	57 ± 15	54 ± 15	56 ± 15
Molecular type [*n* (%)]	IDH-wt	442 (72)	101 (63)	311 (76)
	IDH-mut non-codel	89 (14)	42 (26)	84 (20)
	IDH-mut codel	84 (14)	17 (11)	15 (4)

Abbreviation: SVM, support vector machine.

**Figure 3.**
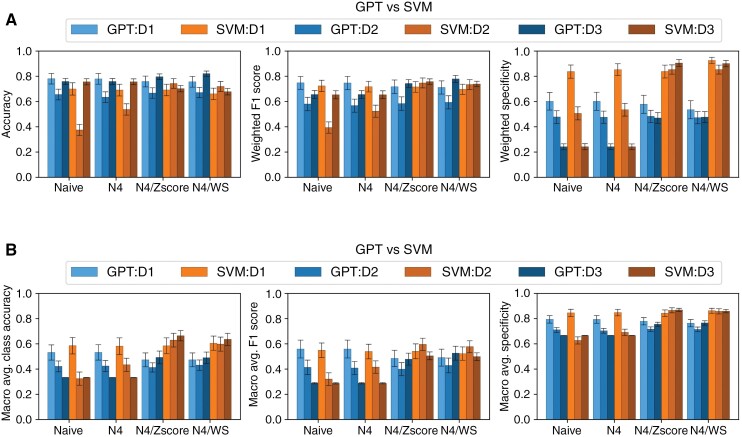
Comparison of (A) weighted and (B) macro averaged accuracy, *F*1-score, and specificity between GPT and SVM models, across different datasets (D1, D2, and D3) and normalization methods (Naive, N4, N4/Zscore, and N4/WS). The error bar represents the SD calculated through bootstrapping. Abbreviation: SVM, support vector machine.

Performance analysis of the models on the D1 test dataset revealed better performance of the GPT-4 model compared with the SVM model in terms of accuracy (accuracy weighted by the frequency of each class) across all normalization methods (*P* < .001 each). The accuracy of the GPT-4 model ranged from 0.757 (95% CI 0.754-0.760) with N4/Zscore and N4/WS normalization to 0.780 (0.777-0.782) with N4 normalization alone. The accuracy of the benchmark SVM model ranged from 0.658 (95% CI 0.655-0.661) with N4/WS to 0.697 (95% CI 0.694-0.7) without normalization (Table 2 and Figure 3).

**Table 2.** Comparison of Accuracy and Macro Accuracy Between GPT and SVM Models, Across Different Datasets (D1, D2, and D3) and Normalization Methods (Naive, N4, N4/Zscore, and N4/WS)

Dataset	Normalization	Model	Accuracy (95% CI)	% of Improvement GPT-4 Over Benchmark	*P* Value GPT vs SVM	Macro Accuracy (95% CI)	% of Improvement GPT-4 Over Benchmark	*P* Value GPT vs SVM
D1	Naive	**GPT**	**0.780 (0.777-0.782)**	11.9	<.0001	0.533 (0.529-0.536)	−8.7	<.0001
		SVM	0.697 (0.694-0.700)			**0.584 (0.579-0.588)**		
	N4	**GPT**	**0.782 (0.780-0.785)**	13.3	<.0001	0.535 (0.532-0.539)	−7.9	<.0001
		SVM	0.690 (0.687-0.692)			**0.581 (0.576-0.585)**		
	N4/Zscore	**GPT**	**0.757 (0.754-0.760)**	9.6	<.0001	0.473 (0.470-0.476)	−19.0	<.0001
		SVM	0.691 (0.688-0.694)			**0.584 (0.580-0.588)**		
	N4/WS	**GPT**	**0.757 (0.754-0.760)**	15.0	<.0001	0.475 (0.472-0.479)	−20.7	<.0001
		SVM	0.658 (0.655-0.661)			**0.599 (0.596-0.603)**		
D2	Naive	**GPT**	**0.656 (0.653-0.658)**	73.1	<.0001	**0.423 (0.420-0.425)**	29.4	<.0001
		SVM	0.379 (0.376-0.381)			0.327 (0.324-0.331)		
	N4	**GPT**	**0.631 (0.629-0.634)**	17.3	<.0001	0.422 (0.419-0.425)	−3.0	<.0001
		SVM	0.538 (0.535-0.541)			**0.435 (0.432-0.438)**		
	N4/Zscore	GPT	0.668 (0.666-0.671)	−10.1	<.0001	0.414 (0.412-0.416)	−34.0	<.0001
		**SVM**	**0.743 (0.740-0.745)**			**0.627 (0.624-0.631)**		
	N4/WS	GPT	0.668 (0.666-0.671)	−7.1	<.0001	0.431 (0.428-0.433)	−27.8	<.0001
		**SVM**	**0.719 (0.717-0.722)**			**0.597 (0.594-0.601)**		
D3	Naive	**GPT**	**0.758 (0.756-0.759)**	0.1	.037	**0.333 (0.333-0.333)**	0.3	<.0001
		SVM	0.757 (0.756-0.758)			0.332 (0.332-0.332)		
	N4	GPT	0.759 (0.758-0.760)	0.4	.384	**0.333 (0.333-0.333)**	0.3	<.0001
		SVM	0.756 (0.755-0.758)			0.332 (0.332-0.332)		
	N4/Zscore	**GPT**	**0.795 (0.793-0.796)**	13.6	<.0001	0.491 (0.488-0.494)	−26.2	<.0001
		SVM	0.700 (0.698-0.701)			**0.665 (0.662-0.667)**		
	N4/WS	**GPT**	**0.820 (0.819-0.821)**	20.9	<.0001	0.492 (0.489-0.494)	−22.5	<.0001
		SVM	0.678 (0.677-0.680)			**0.635 (0.632-0.638)**		

Performance metrics with 95% CIs and *P*-values are included, demonstrating how each model is performed under various conditions. The better-performing model in each metric is highlighted in bold. Abbreviation: SVM, support vector machine.

D2 unveiled variability in model efficacy dependent on normalization. Without normalization, the GPT-4 model achieved an accuracy of 0.656 (95% CI 0.653-0.658), significantly outperforming the benchmark model’s accuracy of 0.379 (95% CI 0.376-0.381). This pattern persisted with the N4 normalization, where GPT-4 recorded an accuracy of 0.631 (95% CI 0.629-634) compared with SVMs 0.538 (95% CI 0.535-0.541). Both models showed an increase in performance when Zscore or WS normalization was applied, with the benchmark model significantly outperforming the GPT-4 model (*P* < .001 each). The GPT-4 model showed an accuracy of 0.668 (95% CI 0.666-0.671) for both normalization approaches compared with an accuracy of 0.743 (95% CI 0.74-0.745) and 0.719 (95% CI 0.717-0.722) of the SVM model based on N4/Zscore and N4/WS normalization, respectively, Table 2 and Figure 3.

Testing on the D3 also showed variability in model efficacy dependent on normalization. While there was no significant difference between the 2 models in N4 normalization (*P* = .38), the accuracy of the GPT4 model increased from 0.759 to 0.795 (95% CI 0.793-0.796) with N4/Zscore and 0.82 (95% CI 0.819-0.821) with N4/WS normalization, significantly outperforming the benchmark model (*P* < .001 each). At the same time, however, the benchmark model showed significantly better macro accuracy (equal weightage to each class irrespective of frequency) than the GPT-4 model with a macro accuracy of 0.665 (95% CI 0.662-0.667) and 0.635 (0.632-0.638) compared with 0.491 (95% CI 0.488-0.494) and 0.492 (0.489-0.494) with N4/Zscore and N4/WS, as shown, respectively, Table 2 and Figure 3.

**Figure 4.**
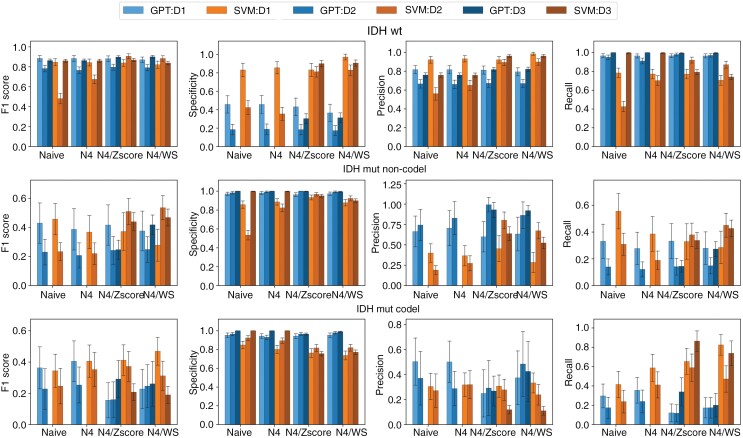
Comparison of performance metrics between GPT and SVM models for the 3 glioma subtypes across different datasets and normalization methods (Naive, N4, N4/Zscore, and N4/WS). Each row represents 1 glioma subtype: IDH-wildtype (IDH-wt), IDH-mutant with 1p/19q-codeletion (IDH-mut codel), and IDH-mutant without 1p/19q-codeletion (IDH-mut non-codel). Each column corresponds to a specific performance metric: sensitivity, specificity, precision, and *F*1-score. The datasets include an internal testing set (D1) and 2 external datasets (D2 and D3). The bars in each plot are color-coded to differentiate the models and datasets. The error bar represents the SD calculated through bootstrapping. Abbreviation: SVM, support vector machine.

A class-wise analysis of the models showed a difference in performance between the glioma molecular subtypes. In the IDH-wt group, GPT-4 models generally demonstrated higher recall rates, with the highest recall for the internal D1 test dataset being 0.967 (95% CI 0.966-0.968) without normalization and for the D2 at 0.981(95% CI 0.980-0.982) with N4/Zscore. The SVM models showed superior precision, especially with N4/Zscore and N4/WS normalization in all 3 datasets. Specificity was consistently lower in GPT-4 models, with the highest for GPT-4 at the HD dataset recorded at 0.462 (95% CI 0.456-0.468) as depicted in Figure 4 and Supplementary Figures 1 and 2.

The performance of GPT-4 was notably lower in the IDH-mut non-codel group. GPT-4 models tested on the D1 demonstrated an *F*1-score of 0.437 (95% CI 0.428-0.445) compared with an *F*1-score of 0.460 (95% CI 0.453-0.467) for SVM models without normalization. Regardless of the normalization approach, recall rates were lower for GPT-4 models in all 3 datasets. Both GPT-4 and SVM models exhibited high specificity ranging from 0.971 (95% CI 0.97-0.973) to 1 (95% CI 1-1) for GPT-4 models and from 0.534 (0.531-0.537) to 0.997 (0.997-0.997) for SVM models as illustrated in Figure 4 and Supplementary Figures 1 and 2.

Analysis of Figure 4 alongside confusion matrices of Supplementary Figures 1 and 2 reveals the lowest performance metrics among glioma types are attributed to the IDH-mut 1p/19q-codel group. The SVM models show the highest *F*1-score of 0.469 (95% CI 0.464-0.475) for D1 with N4/WS normalization compared with an *F*1-score of 0.232 (95% CI 0.224-0.240) of the corresponding GPT-4 model.

## Discussion

Effective ML model development requires mathematics and computer science knowledge, making it challenging for many clinicians to access. In this study, we investigated the ability of the LLM GPT-4 to autonomously develop a radiomics-based ML model to predict different glioma molecular types. We compared its performance against a previously published handcrafted model.^16^ In that prior publication, we also investigated and highlighted the impact of various normalization methods on model performance. We will not re-examine this aspect in the current discussion to maintain clarity and avoid redundancy.

LLMs have shown significant potential in clinical applications, including summarizing patient encounters, generating clinical notes, and providing evidence-based guideline recommendations.^27,28^ By automating and streamlining administrative tasks, LLMs have demonstrated their capacity to enhance operational efficiency and contribute to improved patient outcomes.^29^ Despite these advancements, their application has predominantly focused on text-based data, with limited exploration of their integration with complex quantitative datasets such as radiomics. Overall, most ML models created by GPT-4 within our study exhibited accuracy comparable to or better than the benchmark model, aligning with findings from existing research outside the field of radiomics. Tayebi Arasteh et al. have recently examined the ability of GPT-4 ADA to independently develop ML models utilizing 4 extensive, publicly available clinical datasets from large-scale trials. These models, designed to predict variables such as biomarker presence, cancer progression, and disease complications, based on blood test results, cytologic and epidemiologic as well as gene sequence data, demonstrated performance on par with the benchmark models.^8^ However, examining the class-specific metrics in our study reveals that despite explicit instructions to address class imbalance during training, GPT-4’s performance lagged the benchmark model in classifying IDH-mut non-codel and IDH-mut codel types. A possible reason for this could be the different approaches to managing class imbalance. While the benchmark model utilized a combination of oversampling, specifically, SMOTE, which generates new observations of the minority class by sampling from similar minority class observations, and undersampling of the majority class, GPT-4 relied on class weights.^30^ This approach was necessary because the Python package required for SMOTE was not available in the GPT-4 environment during our experiments. Class weights adjust the loss function by increasing or decreasing the penalty for classes based on their weight. This adjustment increases the model’s focus on underrepresented classes without introducing new data points, making it computationally efficient and less prone to overfitting. However, class weights may not address the lack of sample diversity within minority classes, limiting the model’s capacity to learn varied representations of the minority classes. SMOTE can enhance model sensitivity by creating a more balanced representation of classes, reducing the likelihood of the model underfitting minority classes. However, oversampling techniques can also increase the risk of overfitting, especially in small datasets, as the synthetic samples are derived from existing data points and may lead the model to learn redundant patterns.^30,31^ This difference in handling imbalance could account for the variations in model performance.

GPT-4 significantly enhances the usability of ML by streamlining the development and implementation process. It manages tabular data input, advises on data processing, constructs the model, and delivers results in the desired format. Its ability to interact with users using text-based prompts provides a natural and effective interface for developing ML models while automation simplifies the workflow. However, it is essential to recognize that despite the model’s capacity to streamline access to ML modeling, a solid understanding of the foundational principles of ML remains crucial. This necessity arises because the efficacy and reliability of outputs generated by GPT-4 heavily depend on the quality and precision of the input prompts it receives. Users must have a robust grasp of ML fundamentals to formulate these prompts effectively, design meaningful experiments, and critically evaluate the results. Therefore, while GPT-4 significantly lowers the barrier to entry in deploying ML models, the skill in crafting appropriate prompts and interpreting results cannot be understated, underscoring the indispensable role of foundational knowledge in ML to harness the full potential of such advanced AI technologies. Furthermore, using LLMs in clinical research and practice brings several challenges, including data privacy, security, model interpretability, reliability, and ethical considerations.^32,33^ One of the primary concerns in using LLMs for clinical applications is safeguarding patient data. Patient information, including identifiers such as race, ethnicity, medical history, and other personal details, can be inadvertently disclosed when clinicians interact with LLMs. Although OpenAI’s model development involves continual improvement through data from prior interactions, the lack of transparency around data retention policies and the potential for unintended data persistence heightens privacy concerns. Unlike conventional healthcare software, where data storage and processing are tightly controlled, LLMs operate within dynamic architectures that do not currently support retroactive data deletion. Once information is in an LLM may remain in the model’s ecosystem indefinitely, creating vulnerabilities for data breaches or unauthorized access.^34^ Healthcare data are subject to stringent regulations such as the Health Insurance Portability and Accountability Act (HIPAA) in the United States and the General Data Protection Regulation (GDPR) in the European Union (https://eur-lex.europa.eu/legal-content/EN/TXT/?uri=CELEX:52012PC0011). These regulations mandate strict data handling practices, including data minimization, transparency, and the right to deletion. To address these data privacy and security concerns, healthcare institutions considering LLMs should implement stringent safeguards like robust data anonymization and de-identification protocols before any data is submitted to the model.^35^ For highly sensitive healthcare applications, on-premises deployment of LLMs or the use of privacy-compliant, fine-tuned models may provide an alternative that aligns better with regulatory standards. Hosting the model within a controlled environment allows healthcare providers to maintain complete control over the data pipeline, eliminate dependence on third-party servers, and reduce risks associated with external data handling policies. In addition to privacy and security, LLMs raise concerns about interpretability and reliability. Healthcare professionals must understand how and why an LLM generates specific outputs in clinical settings, significantly if these outputs influence patient care.^36^ Black-box models that lack interpretability can undermine clinician trust, introduce biases, and complicate clinical decision-making. Therefore, explainable AI techniques should be prioritized in LLM implementations to allow clinicians to review the reasoning behind outputs, verify consistency with clinical guidelines, and identify potential biases.^37^ Thus, it is crucial for users to carefully consider the benefits and potential risks of using this tool.

Our study has some limitations: First, the black-box nature of many ML models, including those developed by GPT-4, can be a significant barrier in clinical settings where understanding the decision-making process is crucial. In our study to enhance transparency and oversight, we reimplemented the provided Python code by GPT-4 and checked by an experienced data scientist. Secondly, data cleaning is a crucial and time-intensive preprocessing step before developing ML models. This task was performed by humans and not GPT-4. However, it should be noted that this does not demand the same level of mathematical and computational expertise as required for developing the models themselves.

In conclusion, GPT-4 can autonomously develop radiomics-based ML models without an expert data scientist, achieving performance comparable to handcrafted ML models. However, its poorer class-wise performance due to unbalanced datasets shows limitations in handling complete end-to-end ML pipelines, highlighting the need for critical evaluation of the results and prompts.

## Supplementary Material

vdae230_suppl_Supplementary_Figures

## Data Availability

The Heidelberger data (D1) referenced in this study are proprietary and confidential, belonging exclusively to the Heidelberg University Hospital. Due to the sensitive nature of this information and following our organization’s policies and confidentiality agreements, these data cannot be made available to third parties. In contrast, The Cancer Genome Atlas (TCGA) dataset (D2) and the UCSF (University of California, San Francisco) data (D3) utilized in our research are publicly available.
