# Supplementary material for: The potential of GPT-4 advanced data analysis for radiomics-based machine learning models
Source: Neurooncol Adv. 2024 Dec 23;7(1):vdae230. doi: 10.1093/noajnl/vdae230 (PMC11707530; doi:10.1093/noajnl/vdae230)
Supplement: vdae230_suppl_Supplementary_Figures [file vdae230_suppl_supplementary_figures.docx]

**Supplementary**

**Supplementary Materials and Methods**

*MR acquisition protocol at the executing institute*

Sequence parameters for T1 and cT1 MP-RAGE (3D sagittal or axial) were as follows: TI = 900–1100 ms, TE = 3–4 ms, TR = 1710–2250 ms and FA = 15°; for T2 (2D, axial): TE = 85–88 ms; TR = 2740–5950 ms; section thickness, 5 mm; spacing, 5.5mm; for FLAIR (2D, axial): TI = 2400–2500 ms; TE = 85–135 ms; TR = 8500–10 000 ms; section thickness, 5 mm; spacing, 5.5 mm.

**Supplementary Figure 1.** Confusion matrix generated using SVM classification algorithm across different datasets (D1, D2, D3) and normalization methods (Naive, N4, N4/Zscore, N4/WS). The right axes represent the number of cases for each glioma subtype (n), and the value in cells represents the fraction of “n” predicted as the corresponding label.


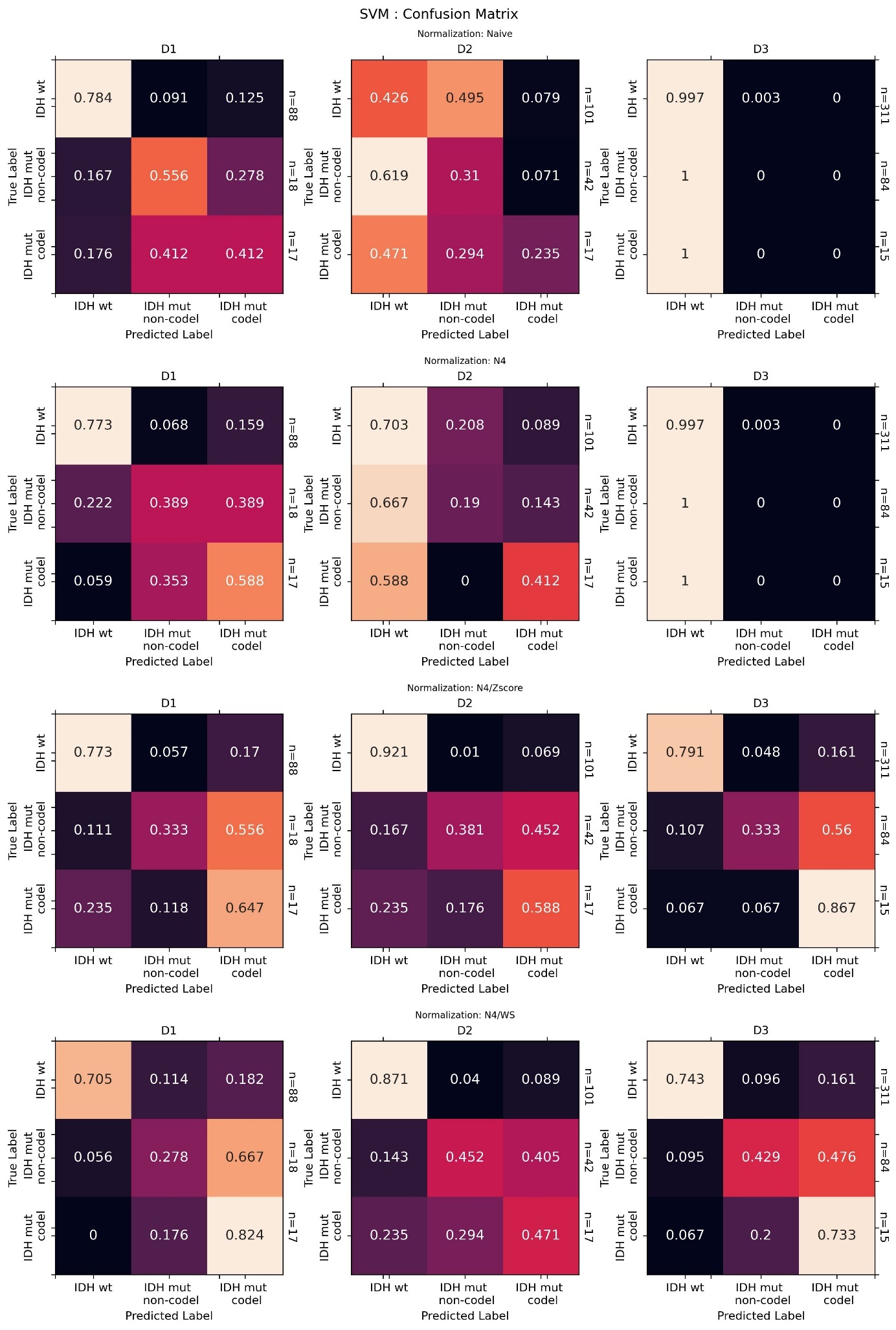


**Supplementary Figure 2.** Confusion matrix generated using GPT trained classification algorithm across different datasets (D1, D2, D3) and normalization methods (Naive, N4, N4/Zscore, N4/WS). The right axes represent the number of cases for each glioma subtype (n), and the value in cells represents the fraction of “n” predicted as the corresponding label.


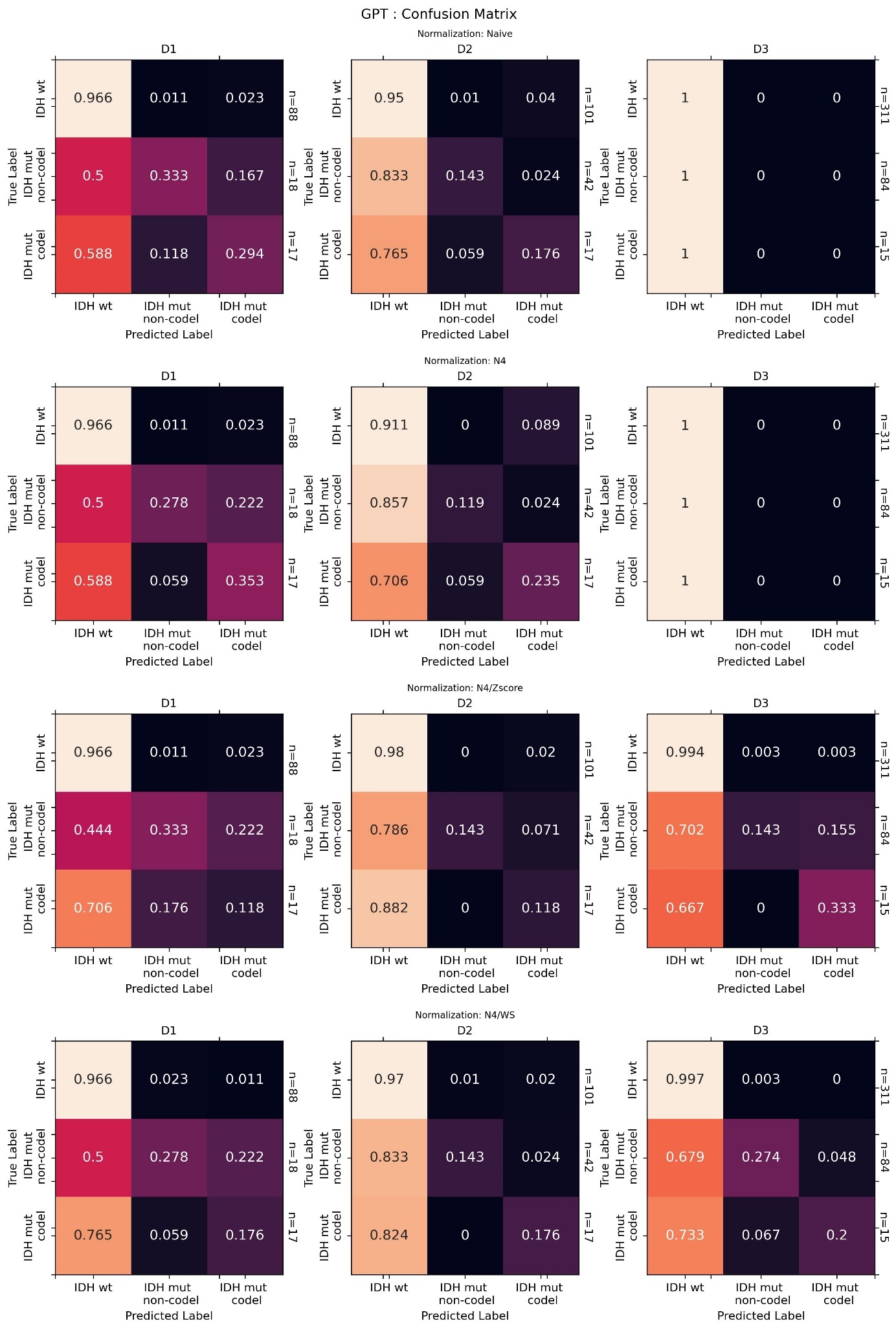


**Supplementary Figure 3.** Screenshot of the prompt used to instruct GPT-4*.

**
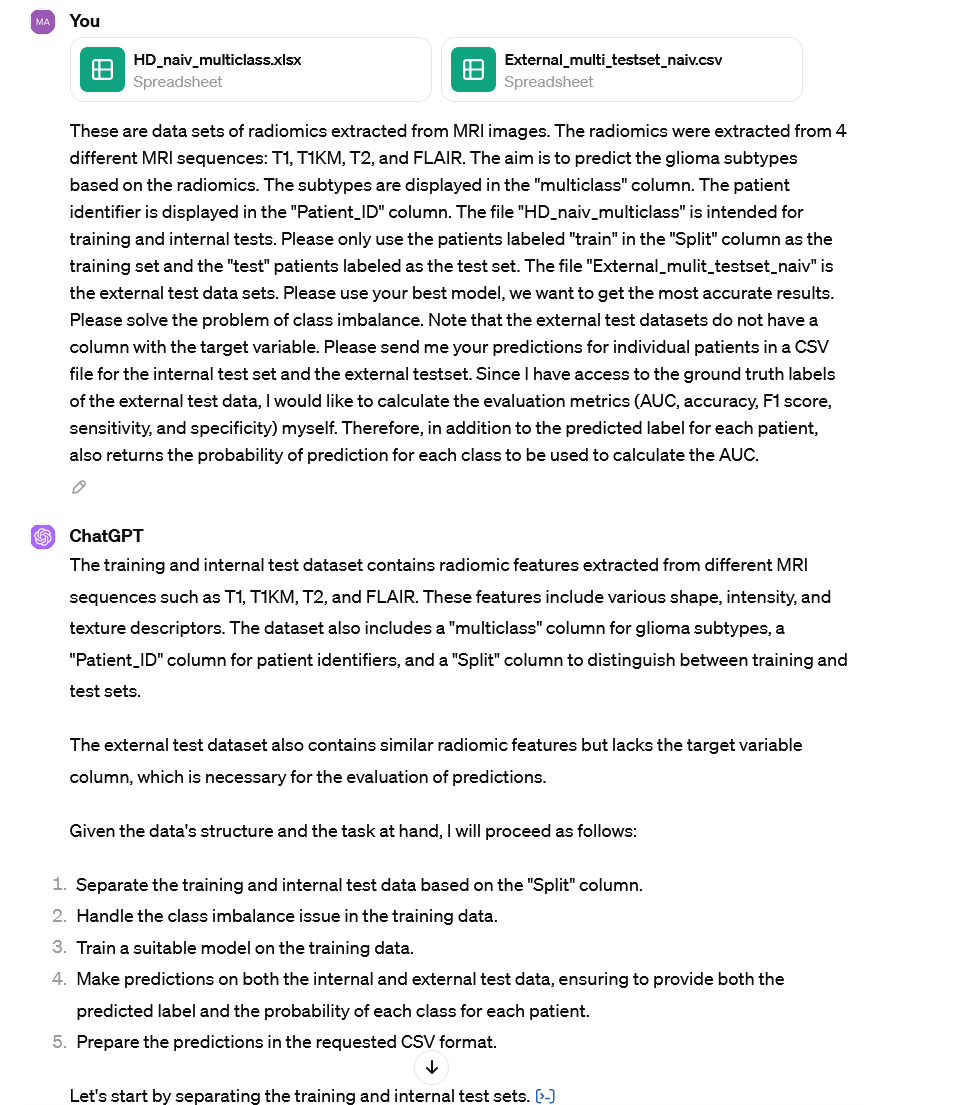
**

* We excluded the Receiver Operating Characteristic (ROC) curve and Area Under the Curve (AUC) metric from the final version of the manuscript due to its limited interpretability and non-standard implementation in multiclass classification, opting instead for precision, specificity, recall, and F1-score metrics to provide more class-specific insights consistent with multiclass classification standards.
